# Meta‐Analysis of COVID‐19 Cluster Events Suggestive of Long‐Distance Airborne Transmission/Inhalation

**DOI:** 10.1002/mbo3.70232

**Published:** 2026-04-02

**Authors:** Mayuko Yagi, Ryoma Tasaki, Jun Komano

**Affiliations:** ^1^ Department of Microbiology and Infection Control Faculty of Pharmacy, Osaka Medical and Pharmaceutical University Osaka Japan

**Keywords:** cluster event, COVID‐19, long‐distance airborne transmission/inhalation, meta‐analysis, SARS‐CoV‐2

## Abstract

SARS‐CoV‐2 spreads through both contact and airborne routes, the latter encompassing airborne transmission/inhalation as well as the direct deposition of infectious respiratory particles. During the early phase of the COVID‐19 pandemic, numerous cluster events were suspected to involve long‐distance airborne transmission/inhalation. We conducted a comparative analysis of these cluster events to characterize outbreak settings and associated clinical parameters. Thirteen cluster events from 2020 attributed to the original SARS‐CoV‐2 strain were examined, including choral activities, indoor sports, and bus tours. Incubation periods and infection–hospitalization rates (IHRs) were compared across settings and against estimates from large‐scale cohort studies that predominantly reflect transmission via direct deposition. Statistical analyses were performed using the Mann–Whitney *U* test, Student's *t*‐test, and Fisher's exact test (*p* < 0.05). The mean incubation period in suspected long‐distance airborne transmission/inhalation cases was 6.1 ± 3.9 days (median: 5 days; *N* = 176), with indoor sports and choral events showing significantly shorter incubation periods (*p* = 0.034). The average IHR was 6.7 ± 12.5%, with significantly higher rates in choral clusters (*p* = 0.013). Age‐adjusted IHRs were lower in long‐distance airborne transmission/inhalation‐related clusters than those reported from contact‐tracing datasets. This analysis provides an integrated evaluation of long‐distance airborne transmission/inhalation settings and their associated clinical characteristics. Activities involving vigorous respiration may contribute to shorter incubation periods and higher disease severity, potentially reflecting increased viral inoculum at exposure.

## Graphical abstract

Comparison across infection settings suggests a potential link between activities involving deep breathing and the clinical course of COVID‐19. Activities involving vigorous respiration may contribute to shorter incubation periods and higher disease severity, potentially reflecting increased viral inoculum at exposure.

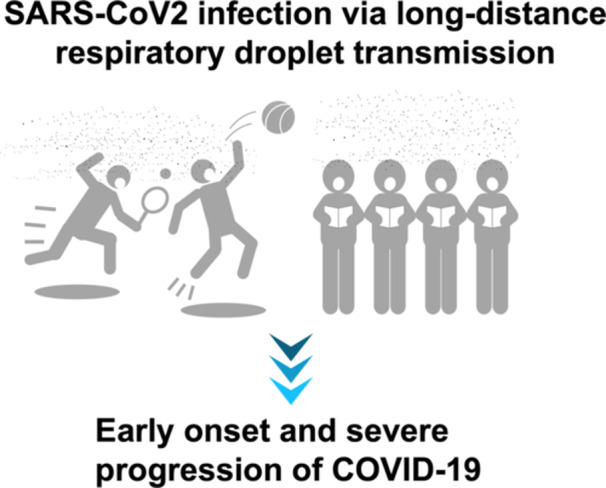

## 1 Introduction

Coronavirus disease 2019 (COVID‐19) is caused by severe acute respiratory syndrome coronavirus 2 (SARS‐CoV‐2) (Wiersinga et al. 2020). The initial outbreak occurred in Wuhan, China, in late 2019 and rapidly spread worldwide. The original virus, often referred to as the Wuhan or ancestral strain, circulated globally until mid‐2020, after which multiple variants emerged.

Accumulated evidence suggests that SARS‐CoV‐2 primarily spreads among humans via virus‐containing respiratory aerosols/droplets or infectious respiratory particles (IRPs) (Tang et al. 2023; Arienzo et al. 2023). Historically, two respiratory transmission modes have been defined: airborne transmission and droplet transmission. Airborne transmission involves the spread of infectious disease through small, pathogen‐laden particles that can remain suspended in the air for hours and be inhaled by susceptible individuals. Such fine respiratory particles can travel more than 2 m from their source (Wang et al. 2021). In contrast, droplet transmission occurs when infected individuals cough, sneeze, or talk, producing large respiratory droplets that typically travel less than 2 m before depositing on the mucosal surfaces of the eyes, nose, or mouth of nearby infectee. In addition to these classical terms, “aerosol transmission” is often used interchangeably with “airborne transmission,” although it lacks a standardized definition in public health. To improve scientific precision and minimize misunderstanding among researchers, the World Health Organization (WHO) proposed updated terminology for IRP‐mediated transmission in April 2024 (ISBN: 978‐92‐4‐008918‐1), introducing the broader concept of “transmission through the air.” This category is subdivided into “airborne transmission/inhalation” and “direct deposition.” The historical term “airborne transmission” corresponds to long‐distance airborne transmission/inhalation, whereas “droplet transmission” corresponds to transmission via “direct deposition.” However, the boundary between these two modes is not absolute and instead overlaps. To maintain clarity, this study consistently uses the term “long‐distance airborne transmission/inhalation,” in accordance with the WHO terminology. Severe acute respiratory syndrome (SARS), caused by a coronavirus closely related to SARS‐CoV‐2, is known to spread via long‐distance airborne transmission/inhalation (Yu et al. 2004). Several other pathogens share this transmission mode, including measles virus, variola virus, and *Mycobacterium tuberculosis* (Wang et al. 2021; Fennelly 2020). Under certain environmental conditions, influenza virus and respiratory syncytial virus (RSV) may spread not only through direct deposition but also via long‐distance airborne transmission/inhalation (Wang et al. 2021).

After exposure to a pathogen, there is a period before clinical symptoms develop, known as the incubation or latency period, also referred to as the asymptomatic interval. Cohort studies estimate the incubation period of COVID‐19 to be primarily between 3 and 6 days (Xin et al. 2022; Ma et al. 2022; Galmiche et al. 2023; Zhao et al. 2021). Asymptomatic carriers can unknowingly shed and transmit the virus, making it difficult to determine the exact date of exposure for infectees (Arons et al. 2020; He et al. 2020; Furukawa et al. 2020). The latency period has often been investigated through contact tracing, where patients report the timing of symptom onset and their contact with infected individuals. Most contact tracing reports likely reflect droplet or contact transmission, because long‐distance airborne transmission/inhalation is typically unnoticeable to infectees (Wu et al. 2022; Zeng et al. 2023).

One factor influencing the latency period is the viral strain. The Omicron variant of SARS‐CoV‐2 has been associated with a shorter incubation period than earlier variants (Galmiche et al. 2023; Wu et al. 2022; Zeng et al. 2023). Additional factors, such as age, gender, and underlying health status, have also been reported to affect the incubation period and disease severity (Galmiche et al. 2023; Guallar et al. 2020; Ogata and Tanaka 2023). Although this has not been directly verified in human challenge experiments, it has been proposed that the initial viral load plays a role in determining the length of the incubation period and the disease severity (Guallar et al. 2020). However, previous studies have not systematically examined whether the setting of infection influences these outcomes. To address this, we focused on SARS‐CoV‐2 infections suspected of long‐distance airborne transmission/inhalation, which have been reported across a variety of cluster settings.

From a public health perspective, long‐distance airborne transmission/inhalation is suspected when three conditions are met: (1) other transmission routes, such as direct deposition and direct contact, can be reasonably ruled out (e.g., the infector and infectee were never closer than hand‐reach distance); (2) the ventilation at the cluster event site is poor and/or airflow patterns reasonably explain the infector–infectee transmission (e.g., the infector was positioned upwind of the infectee); and (3) the exposure opportunity is limited to the day of the spreading event (e.g., the infectious disease is not widely prevalent in the community). Several epidemiological studies have reported SARS‐CoV‐2 cluster events that likely involved long‐distance airborne transmission/inhalation. In this study, we investigated individuals infected with SARS‐CoV‐2 via long‐distance airborne transmission/inhalation and assessed the relationship between cluster settings and clinical parameters, specifically the incubation period and the hospitalization rate.

## 2 Materials and Methods

### 2.1 Meta‐Analysis of Outbreaks Suspected of Long‐Distance Airborne Transmission/Inhalation of SARS‐CoV‐2

A literature search was conducted using PubMed and internet search engines to identify articles of COVID‐19 cluster events investigated by epidemiologists that suggested possible involvement of long‐distance airborne transmission/inhalation. A total of 22 cluster event reports were identified. Among these reports, point‐source outbreaks were selected for incubation period analysis, in which exposure to SARS‐CoV‐2 was limited to a single day and symptom onset was clearly documented. In addition, only cluster events attributed to the original Wuhan strain were included in the analysis, as incubation periods vary among virus strains (Galmiche et al. 2023). Ultimately, 12 events were selected for incubation period analysis (cluster events #1‐12, Table 1) (Lu et al. 2020; Li et al. 2021; Barani et al. 2021; Shen et al. 2020; Toyokawa et al. 2022; Brlek et al. 2020; Hamner et al. 2020; Charlotte 2023; Ou et al. 2022; Kwon et al. 2020; Katelaris et al. 2021; Hwang et al. 2021; Pauser et al. 2021). The incubation period was defined as the time from virus exposure to the onset of symptoms. Index cases were excluded from the analysis. In instances where the index case could not be determined due to the absence of symptoms on the day of the cluster event, the first symptomatic individual in the cluster was designated as the index case. Exclusion of the index case was not performed for cluster event #12, since the detailed case information was lacking (Pauser et al. 2021). The incubation period of the secondary cases (*N* = 216) was analyzed. Among the secondary infections, 176 cases were included in the case‐based incubation period analysis after excluding four cases as outliers, as determined by the Smirnov‐Grubbs test. For the infection‐hospitalization rate (IHR) analysis, 13 cluster events were analyzed (Table 1). When the cluster event occurred in a hospital, only healthcare providers—excluding hospitalized patients—were included (cluster event #8, Table 1) (Ou et al. 2022). Cluster event #13, occurred in a factory, was also included in this analysis, as precise specification of the exposure date was not essential for evaluating IHR analysis (Günther et al. 2020). The quality assessment of cluster events is provided in the supplementary data (Table S1).

**Table 1:** COVID‐19 cluster events suspected to involve long‐distance airborne transmission/inhalation.

ID^a^	Location^b^	Setting	Event date (month/year)	Number of econdary cases (N)^c^	Incubation period (days, median)	Index case status^d^	Hospitalized (N)	Infection‐hospitalization rate (%)	Reference
1	Guangzhou, China	Restaurant	01/2020	9	7	S	0	0.0%	18, 19
2	Hunan Province, China	Bus tour	01/2020	9	4	S	0	0.0%	20
3	Zhejiang province, China	Bus tour	03/2020	32	8	AC	0	0.0%	21
4	Okinawa, Japan	Domestic flight	03/2020	16	8	S	1	6.3%	22
5	Maribor, Slovenia	Indoor sports	03/2020	4	4	S	0	0.0%	23
6	Washington, USA	Choral singing	03/2020	52	6	S	3	5.8%	24
7	France	Choral singing	03/2020	17	5	AC	7	41.2%	25
8	Chennai, India	Hospital	04/2020	19 (6)	10	S	0	0.0%	26
9	Jeonju, Korea	Restaurant	06/2020	2	5.5	AC	0	0.0%	27
10	Sydney, Australia	Choral singing	07/2020	12	5	S	3	25.0%	28
11	Seoul, South Korea	Apartment	08/2020	8	5.5	S	0	0.0%	29
12	Germany	Indoor sports	11/2020	36	4	AC or S	3	8.3%	30
13	Rheda‐Wiedenbruck, Germany	Factory	05/2020	36	—	S	0	0.0%	31
	Average (Total)	—	—	19.4 (252)	6.0	—	1.4	7.2%	—

^a^ Cluster events #1–12 were included in the incubation period analysis. Cluster event #13 was included only in the infection–hospitalization rate (IHR) analysis because the exact exposure date could not be determined.

^b^ Only the country is indicated unless a specific geographic location was reported in the original literature.

^c^ For cluster events occurring in healthcare settings, only healthcare workers were included in the IHR analysis; hospitalized patients were excluded to avoid confounding by disease severity at baseline.

^d^ S, symptomatic; AC asymptomatic carrier.

### 2.2 Statistical Analysis

The Mann‐Whitney *U* test, Student's *t*‐test, Smirnov‐Grubbs test, and Fisher's exact test were used to assess statistical differences, as appropriate. The Smirnov‐Grubbs test was applied to identify and eliminate extreme values. To identify outliers, the value farthest from the mean was examined using the Smirnov‐Grubbs test. After removing an identified outlier, the same test was repeatedly applied to the remaining dataset. The incubation period of each case is shown in the supplementary data (Table S2). A *p*‐value less than 0.05 was considered statistically significant.

## 3 Results

### 3.1 Overview of the SARS‐CoV‐2 Cluster Events Suspected of Long‐Distance Airborne Transmission/Inhalation

Among 22 reported COVID‐19 cluster events in which long‐distance airborne transmission/inhalation was suspected, this study focused on 13 point‐source outbreaks caused by the original SARS‐CoV‐2 strain that occurred between January and November 2020 (cluster events #1–13, Table 1). These clusters were reported from eight countries. All events took place in indoor settings. For incubation period assessment, 12 cluster events were analyzed. A total of 216 individuals were secondarily infected, with an average of 18.0 cases per cluster (range: 2–52). In 8 of the 12 clusters, the outbreak was initiated by a symptomatic index case. For the assessment of the IHR, 13 cluster events were included, comprising the 12 events used in the incubation period analysis and one additional cluster (cluster event #13) where the date of exposure could not be clearly identified.

### 3.2 Analysis of the Incubation Period

**Figure 1:**
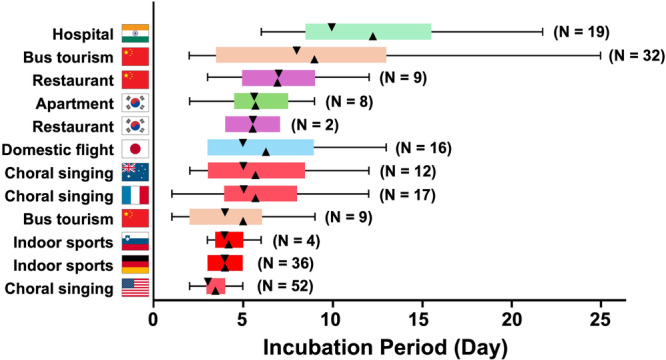
Incubation periods of SARS‐CoV‐2 infection in clusters suspected of long‐distance airborne transmission/inhalation. Box‐and‐whisker plots showing the incubation periods of SARS‐CoV‐2 infections from 12 cluster events suspected of long‐distance airborne transmission/inhalation, sorted in descending order of median incubation period. The vertical axis displays the event type and country, while the horizontal axis represents the incubation period in days. The downward triangle within each box denotes the median; the upward triangle indicates the mean; box limits correspond to the 25th and 75th percentiles; whiskers extend to 1.5 times the interquartile range. The sample size for each cluster is shown in parentheses. Events involving indoor sports or choral singing are highlighted in red. For details of each event, see Table 1.

In the event‐based analysis, the median of the incubation period of the 12 cluster events was 5.0 days (95%CI 3.9–6.1). Notably, incubation periods for indoor sports and choral activities were significantly shorter than those for other settings (*p* = 0.034 by Mann‐Whitney *U* test, Figure 1). The two choral events from the United States (Hamner et al. 2020) and France (Charlotte 2023) involved older participants, with median ages of 69 and 66.9 years, respectively. The ages of infected individuals in the other cluster events were not fully reported (Table S1). Although longer incubation periods have been associated with older age (Galmiche et al. 2023; Tan et al. 2020), these two choral events exhibited some of the shortest incubation periods among the 12 clusters. This suggests that factors other than age contributed to the shortened incubation periods observed in choral activity–related clusters. One likely factor is the nature of the activity itself, specifically activities involving deep breathing, which may have contributed to accelerated symptom onset.

Incubation periods from large‐scale contact tracing studies were compared with those from cluster events suspected of long‐distance airborne transmission/inhalation (Table 2). Contact tracing identifies individuals who have been in contact with symptomatic patients; therefore, most cases are presumed to result primarily from direct deposition or contact transmission. This is partly because infectees are unlikely to notice exposure to IRPs via long‐distance airborne transmission/inhalation. A meta‐analysis of 119 cluster events involving the original strain of SARS‐CoV‐2 reported a median incubation period of 6.7 days (95% CI: 6.3–7.0) (Wu et al. 2022). Another meta‐analysis of 24 cluster events reported a median of 5.8 days (McAloon et al. 2020). The authors pointed out that contact tracing often lacks accuracy in determining the exact date of virus exposure. When their analysis was restricted to cases with clearly defined exposure dates, the median incubation period was 5.1 days (McAloon et al. 2020). These findings suggest that, in cluster‐based meta‐analyses, the incubation periods of COVID‐19 associated with long‐distance airborne transmission/inhalation are not substantially different from those identified through contact tracing.

**Table 2:** Incubation period estimates for COVID‐19 using different analytical approaches.

Analytical approach	Metrics	Reference	Data	Incubation period^a^	Comments^b^
Cluster‐based analysis	Median		Cluster events	Median (days, 95%CI)	
		This study	12	5.0 (3.9–6.1)	
		McAloon, et al., *BMJ Open*, 2020	24	5.1 (4.5–5.8)	Higher precision
		McAloon, et al., *BMJ Open*, 2020	24	5.8 (5.0–6.7)	
		Wu, et al., JAMA Network Open, 2022	119	6.7 (6.3–7.0)	
Case‐based analysis	Median		Cases	Median (95%CI)	
		This study	180	5.0 (4.3–5.7)	
		Guan, et al., *NEJM*, 2020	1099	4.0 (2.0–7.0)	
		Denis, et al., *J Med Internet Res*, 2021	1676	4.0 (3.0–5.0)	
		Galmiche, et al., *Lancet*, 2023	7539	4.0 (not provided)	
		Nie, et al., *J Infect Dis*, 2020	2907	5.0 (2.0–8.0)	
	Average		Cases	Average ± SD (days)	
		This study	176	6.1 ± 3.9	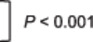
		Galmiche, et al., *Lancet*, 2023	7539	4.6 ± 2.2	

^a^ CI, confidence interval; SD, standard deviation.

^b^ Statistics by Student's *t*‐test.

A comparison was performed against studies conducting case‐based analyses using datasets including more than 1,000 individuals (Galmiche et al. 2023; Denis and Krakowski 2021; Guan et al. 2020; Nie et al. 2020). Across these large‐scale studies, the median incubation period consistently ranged from 4 to 5 days, closely aligning with the values observed in cluster events suspected of long‐distance airborne transmission/inhalation (Table 2).

**Figure 2:**
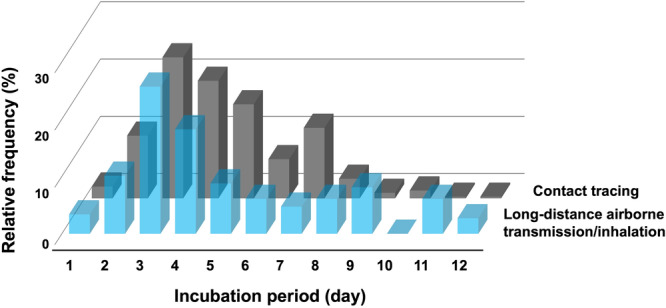
Distribution of incubation periods in SARS‐CoV‐2 infections by transmission mode. Comparison of the incubation period distributions between the long‐distance airborne transmission/inhalation group (blue) and the contact tracing group from a large French cohort (grey; Galmiche et al., *Lancet Microbe* 2023). The vertical axis indicates the proportion of individuals (%) with each incubation period, and the horizontal axis shows the number of days from exposure to symptom onset. Both groups peak on day 3; however, the long‐distance airborne transmission/inhalation group displays a broader distribution, with a greater proportion of cases showing longer incubation periods.

Since the mean is more sensitive to data variability than the median, average values were also examined. In the case‐based analysis, the mean ± standard deviation of the incubation period were 6.1 ± 3.9 days among 176 infectees in long‐distance airborne transmission/inhalation‐associated clusters for whom data was available. In the largest cohort study from France, which included 7539 patients, the mean incubation period was 4.6 ± 2.2 days (Galmiche et al. 2023). This value was significantly shorter than that observed in long‐distance airborne transmission/inhalation‐associated outbreaks (*p* < 0.001, Student's *t*‐test, two‐sided). Visualization of symptom onset timing indicated that both groups peaked at day 3; however, a greater proportion of patients in the airborne group developed symptoms at later time points (Figure 2). This explains why the difference in average incubation periods was statistically significant, even though the medians appeared similar.

### 3.3 Analysis of the Infection‐Hospitalization Rate (IHR)

To assess clinical severity, the IHR was examined, as hospitalization was the most consistently documented indicator of disease severity across cluster event reports. A total of 13 cluster events were included (Table 1). For clusters occurring in hospital settings, only healthcare providers were included, while hospitalized patients were excluded from the analysis to avoid confounding.

**Figure 3:**
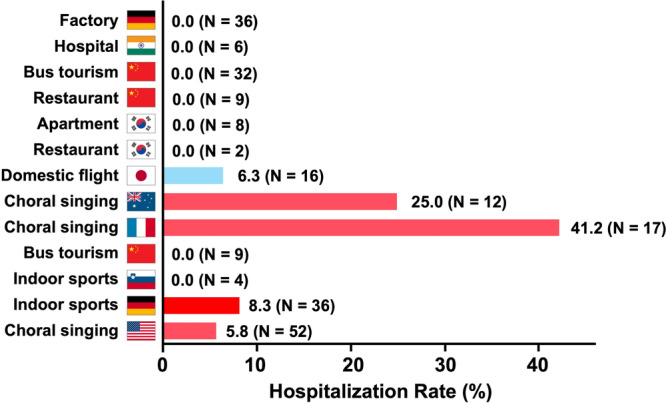
Infection‐hospitalization rates in COVID‐19 clusters suspected of long‐distance airborne transmission/inhalation. The infection‐hospitalization rate (%) is shown for each COVID‐19 cluster event suspected of long‐distance airborne transmission/inhalation. Events are listed in the same order as in Figure 1, with the exception of the German factory cluster event, which is placed at the top. The exact infection‐hospitalization rate is indicated at the end of each bar, with the number of infected individuals shown in parentheses. Color coding is consistent with Figure 1, with choral and indoor sports events highlighted in red. For further details, refer to Table 1.

In the cluster‐based analysis, the mean IHR was 6.7 ± 12.5% (range: 0%–41.2%, *N* = 13). When calculated based on individual patient data, 17 of 239 infected individuals were hospitalized, yielding an IHR of 7.1% (Figure 3). Clusters with shorter incubation periods tended to show higher IHRs. In particular, the IHR was significantly higher in the chorus and indoor sports clusters (16.1%, *N* = 5) compared to other settings (0.78%, *N* = 8; *p* = 0.013, Mann–Whitney *U* test).

The IHR generally increases with age, in contrast to the incubation period, which tends to be longer in older individuals. To address this, cluster events were examined in which participant age information was available. In the French choir cluster, the median age of participants was 66.9 years (Charlotte 2023), while in the US choir cluster, the median age was 69 years old (Hamner et al. 2020). The median ages of the hospitalized cases were 70.5 and 69 years old. The corresponding IHRs were 41.2% and 5.8%, respectively. Combining these two choir events, 10 out of 69 individuals were hospitalized, yielding a pooled IHR of 14.5%. In contrast, two studies based on US nationwide reports from March 2020 estimated IHRs of 27.9% and 28.2% for individuals aged over 50 and 60 years, respectively (Reese et al. 2021; Stokes et al. 2020). Thus, when age was accounted for, IHRs associated with long‐distance airborne transmission/inhalation clusters were significantly lower than those reported in contact‐tracing data mostly representing transmission via direct deposition (*p* = 0.015 and *p* = 0.015, respectively, Fisher's exact test, two‐sided).

## 4 Discussion

COVID‐19 is currently the only respiratory infectious disease for which more than 20 cluster events have been reported with strong epidemiological evidence suggestive of long‐distance airborne transmission/inhalation. Most of these reports investigated cluster events that occurred in 2020, during the early phase of the pandemic. This intense focus on transmission routes reflected the urgent need to identify effective countermeasures.

Our meta‐analysis of point‐source cluster events associated with suspected long‐distance airborne transmission/inhalation of SARS‐CoV‐2 showed that indoor sports and choral activities were associated with earlier symptom onset and higher IHRs, consistent with previous reports linking incubation period to disease progression (Lai et al. 2020). These activities typically involve deep or forceful breathing, which may create conditions favorable for long‐distance airborne transmission/inhalation. In particular, deep breathing has been shown to increase aerosol generation from the alve.

Olar regions—where SARS‐CoV‐2 replicates—by nearly 100‐fold compared with normal breathing (Morawska et al. 2022). The shortened incubation period and elevated hospitalization rate observed in these settings likely reflect a higher viral inoculum at the time of exposure.

In transmission through direct deposition or short‐distance airborne transmission/inhalation, the IRPs are larger in diameter compared to those involved in long‐distance airborne transmission/inhalation. Consequently, these larger IRPs are expected to carry a higher viral load. Therefore, direct deposition and short‐distance airborne transmission/inhalation are theoretically associated with shorter incubation periods and a higher IHR than long‐distance airborne transmission/inhalation. In our comparative analysis, the median incubation periods were similar between these groups. However, when using average values from large cohort studies, long‐distance airborne transmission/inhalation was associated with a longer incubation period. Similarly, age‐adjusted IHRs were consistently lower in long‐distance airborne transmission/inhalation cases. These findings are consistent with the hypothesis that the clinical severity of COVID‐19 correlates with the viral inoculum received at exposure.

## 5 Limitations

The incubation period is influenced by various factors, including viral strain, age, gender, underlying health conditions, and vaccination status (Galmiche et al. 2023; Wu et al. 2022; Zeng et al. 2023; Guallar et al. 2020; Ogata and Tanaka 2023). However, our meta‐analysis was unable to examine these variables in detail, as such information was often missing in the original reports. The viral strain could not be assessed because the circulating virus was predominantly the original strain during the early to mid‐2020 period, when the reported cluster events occurred. This period also preceded the development of COVID‐19 vaccines. Detailed case information was not fully provided in the cluster reports due to ethical considerations aimed at preventing the identification of infected individuals, especially during the early phase of the COVID‐19 pandemic. Under these constraints, our analysis may be subject to residual bias. For example, although choral participants tended to be older, they may have had fewer underlying comorbidities than the general elderly population, which could have influenced clinical outcomes.

When comparing the incubation period and IHR between the long‐distance airborne transmission/inhalation group and the contact tracing group, the difference was more pronounced for IHRs. A human challenge study using intranasal inoculation of the Wuhan strain at a dose of 10 TCID₅₀ reported that many challenged individuals experienced some symptoms as early as day one (Killingley et al. 2022). This finding suggests that retrospective contact tracing may fail to capture cases with mild or subclinical symptoms. Many large cohort‐based datasets rely on self‐reported information collected via telephone interviews or participant registration systems. As such, data accuracy may be influenced by recall bias, the social context of the participants, the manner in which interviewers frame questions, and how responses are interpreted by investigators. Such underreporting represents a general limitation of contact tracing methodologies. These factors may introduce variability that differs from cluster investigations, which typically involve a limited number of cases examined in a more intensive and focused manner. In contrast, hospitalization is typically based on objective clinical findings and is thus considered a more reliable outcome measure. The consistently lower IHRs observed in the long‐distance airborne transmission/inhalation group may provide stronger evidence that the viral load upon exposure was lower in these cases. A similar trend has also been observed in studies of influenza (Paulo et al. 2010; Price et al. 2015). A previous study proposed a possible relationship between exposure dose and disease severity based on a limited number of clusters (Guallar et al. 2020). Direct validation of such an association would require human challenge studies, which are constrained by ethical limitations. The present study provides systematic, although indirect, evidence supporting this relationship. Even within contact‐tracing datasets, more detailed classification of transmission settings could further elucidate correlations between clinical outcomes and transmission routes.

Airborne transmission/inhalation and direct deposition often overlap, making it challenging to distinguish between them in epidemiological investigations. Long‐distance airborne transmission/inhalation is typically inferred rather than directly proven, and it is therefore difficult to establish this mode of transmission with certainty. Among the cases categorized as long‐distance airborne transmission/inhalation, some cases may have actually involved direct deposition or contact transmission. Conversely, although contact‐tracing data are generally assumed to represent transmission via direct deposition or contact transmission, it is likely that a proportion of these cases also involved airborne spread. Despite this potential overlap, we still observed measurable differences in clinical outcomes, suggesting that SARS‐CoV‐2 infections acquired through long‐distance airborne transmission/inhalation may follow a distinct clinical course compared with those transmitted through direct deposition or contact transmission.

## 6 Conclusion

Comparison across infection settings suggests a potential link between activities involving deep breathing and the clinical course of COVID‐19. Clusters attributed to long‐distance airborne transmission/inhalation appear to involve a lower viral inoculum, potentially contributing to milder disease progression.

## Author Contributions

**Mayuko Yagi:** data curation, formal analysis, investigation, writing – review and editing. **Ryoma Tasaki:** data curation, formal analysis, investigation, and writing – review and editing. **Jun Komano:** conceptualization, data curation, formal analysis, investigation, supervision, writing – original draft, and writing – review and editing.

## Funding

The authors received no specific funding for this work.

## Ethics Statement

The authors have nothing to report.

## Conflicts of Interest

The authors declare no conflicts of interest.

## Supporting information


**Table S1:** Quality assessment of epidemiological investigations of COVID‐19 cluster events suspected of long‐distance airborne transmission/inhalation.

**Table S2:** Frequency distribution of incubation periods among cases in COVID‐19 cluster events suspected of long‐distance airborne transmission/inhalation.

## Data Availability

The data that support the findings of this study are openly available in PubMED at https://pubmed.ncbi.nlm.nih.gov.
